# Stability or Plasticity? – A Hierarchical Allostatic Regulation Model of Medial Prefrontal Cortex Function for Social Valuation

**DOI:** 10.3389/fnins.2020.00281

**Published:** 2020-03-31

**Authors:** Hackjin Kim

**Affiliations:** Department of Psychology, Korea University, Seoul, South Korea

**Keywords:** thalamic reticular nucleus, insula, allostasis, interoception, decision-making, prosociality, self-enhancement

## Abstract

The medial prefrontal cortex (mPFC) has long been recognized as the key component of the neurocircuitry involved in various social as well as non-social behaviors, however, little is known regarding the organizing principle of distinctive subregions in the mPFC that integrates a wide range of mPFC functions. The present study proposes a hierarchical model of mPFC functionality, where three functionally dissociable subregions, namely, the ventromedial prefrontal cortex (vmPFC), rostromedial prefrontal cortex (rmPFC), and dorsomedial prefrontal cortex (dmPFC), are differentially involved in computing values of decision-making. According to this model, the mPFC subregions interact with each other in such a way that more dorsal regions utilize additional external sensory information from environment to predict and prevent conflicts occurring in more ventral regions tuned to internal bodily signals, thereby exerting the hierarchically organized allostatic regulatory control over homeostatic reflexes. This model also emphasizes the role of the thalamic reticular nucleus (TRN) in arbitrating the transitions between different thalamo-cortical loops, detecting conflicts between competing options for decision-making, and in shifting flexibly between decision modes. The hierarchical architecture of the mPFC working in conjunction with the TRN may play a key role in adjusting the internal (bodily) needs to suit the constraints of external (environmental) variables better, thus effectively addressing the stability-plasticity dilemma.

## Introduction

Imagine you are an international student who came to study in a country that is culturally very different from that you grew up. How would you maintain stable codes of conduct while updating other codes in a novel social situation? The problem of acquiring new knowledge without disrupting the existing knowledge, the so-called stability-plasticity dilemma, is one of the major obstacles encountered by any adaptive agent (Grossberg, 2013). The human brain is known to be one of the most successful systems for dealing with the stability-plasticity dilemma. Multitudes of researchers in a wide range of academic disciplines, from computer scientists to neuroscientists, have extensively investigated the way the human brain resolves this dilemma. Our knowledge on the exact mechanisms for such a capacity, particularly under social contexts, remains limited.

This article suggests that such a stability-plasticity dilemma can be effectively addressed by the brain’s capacity to predict and prevent homeostatic imbalance, which is called *allostasis* (Sterling and Eyer, 1988; McEwen and Stellar, 1993; Schulkin, 2003), to maintain a state of homeostasis. For example, the brain constantly seeks the optimal regulation of bodily homeostasis by adding increasing amounts of external sensory inputs (e.g., visual, auditory, tactile stimuli) in order to predict and prevent anticipated homeostatic imbalance as early and accurately as possible (*External valuation*). As a result, the most parsimonious pattern of external inputs that led to a successful prediction and prevention of homeostatic imbalance becomes associated with a specific coordinated pattern of somatic or visceral reflexes or both, which is then engaged in a reflex-like fashion whenever the same or a similar input pattern is presented (*Internal valuation*). Importantly, when internal valuation fails to achieve the state of homeostasis, then external valuation will be engaged again to update the internal valuation. This internal-external valuation cycle may be at the heart of the allostatic regulation and also likely to reflect how the brain deals with the stability-plasticity dilemma.

To illustrate how such an allostatic regulation can be linked to social valuation, this article first reviews recent findings of the roles of the medial prefrontal cortex (mPFC) in decision-making in social situations. Next, the *hierarchical allostatic regulation model of the mPFC function* for computing values of social decision-making is proposed, with an emphasis on the three functionally and anatomically dissociable subregions of the mPFC: the ventromedial prefrontal cortex (vmPFC), the rostromedial prefrontal cortex (rmPFC), and the dorsomedial prefrontal cortex (dmPFC). In this model, the mPFC subregions are organized such that more ventral and more dorsal regions are involved in internal and external valuation, respectively, and the intermediate areas are functionally and spatially graded concerning such dimensions. More ventral regions are involved in intuitive value computation to meet internal needs, prioritizing stability, whereas more dorsal regions are involved in deliberative value computation to utilize external information, prioritizing plasticity. External valuation in more dorsal regions is engaged to resolve a conflict that occurs when mutually competing units are simultaneously activated in more ventral regions. Following repeated engagements, such external valuation can serve to update internalized values encoded in the ventral regions. Based on all of these properties above, this model can efficiently address the stability-plasticity dilemma and why such functionality is critical for adaptive behavior in constantly changing social situations.

This review includes literatures from human as well as non-human research including monkeys and rats. Despite some evidence for remarkable cross-species homology among rats, macaques, and humans in the anatomy of the medial prefrontal cortex (Vogt et al., 2013), attention should be paid to the interpretation of the cross-species comparisons reported in the present study regarding functional differences among different subregions of the medial prefrontal cortex.

## Anatomically and Functionally Dissociable Subregions in the Medial Prefrontal Cortex

### Anatomical Boundaries Between the Medial Prefrontal Cortex Subregions

**FIGURE 1:**
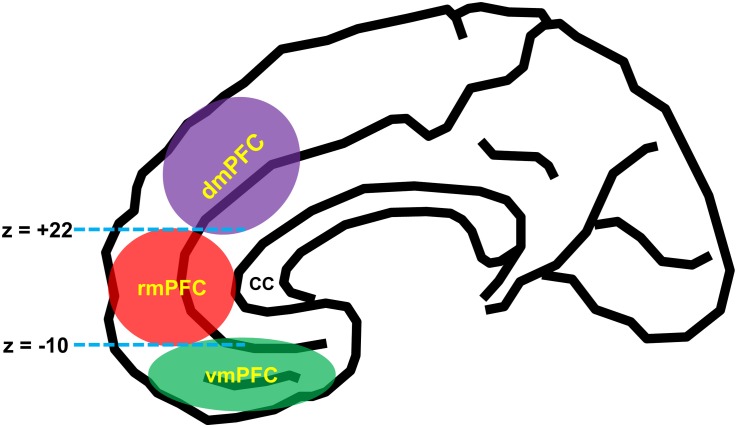
Schematic diagram of anatomical segregation within the medial prefrontal cortex. mPFC can be broadly divided into three functionally and anatomically dissociable subregions: the ventromedial prefrontal cortex (vmPFC) [roughly corresponds to the medial aspect of Brodmann area (BA 11, BA 12, BA 14, and BA 25)], the dorsomedial prefrontal cortex (dmPFC) [BA 9, BA 24 (the pregenual anterior cingulate cortex), and BA 32 (the anterior midcingulate cortex)], and the rostromedial prefrontal cortex (rmPFC) [BA 10, BA 24 (the pregenual anterior cingulate cortex), and BA 32 (the pregenual anterior cingulate cortex)]. The dmPFC and rmPFC are divided by the z-plane of +22, and the rmPFC and vmPFC are divided by the z-plane of –10 (Lieberman et al., 2019). cc, corpus callosum.

According to the influential anatomical studies (Vogt, 2005; Mackey and Petrides, 2014; Joyce and Barbas, 2018; Palomero-Gallagher et al., 2019), human mPFC can be broadly divided into three functionally and anatomically dissociable subregions: (1) the vmPFC [roughly corresponds to the medial aspect of Brodmann area (BA) 11, BA 12, BA 14, and BA 25], (2) dmPFC [BA 9, BA 24 (the pregenual anterior cingulate cortex), and BA 32 (the anterior midcingulate cortex)], and (3) rmPFC [BA 10, BA 24 (the pregenual anterior cingulate cortex), and BA 32 (the pregenual anterior cingulate cortex)]. For a practical purpose, a recent neuroimaging study utilized the MNI coordinate system such that the vmPFC and rmPFC are divided by the z-plane of –10 (MNI coordinate system), and the dmPFC and the rmPFC are divided by the z-plane of +22 (Lieberman et al., 2019; Figure 1).

It is well-known that these subregions have unique patterns of anatomical (Haruno and Kawato, 2006; Yin and Knowlton, 2006; Haber and Knutson, 2010) as well as functional (Bzdok et al., 2013; de la Vega et al., 2016) connections with other neural structures. More specifically, recent meta-analyses of functional connectivity revealed that the vmPFC is functionally connected with the nucleus accumbens, amygdala, and thalamus; the rmPFC with the nucleus accumbens, hippocampus, posterior cingulate cortex, and retrosplenial cortex; and the dmPFC with the inferior frontal gyrus, temporo-parietal junction, and middle temporal gyrus (Bzdok et al., 2013; de la Vega et al., 2016). It should be noted, however, that these meta-analyses may not reflect the full connectivity because they are based on fMRI studies with limited spatial and temporal resolution.

The mPFC subregions do not seem to be mutually independent, but functionally inter-connected with each other. For example, it has been proposed that reinforcement learning occurs through multiple mutually interacting cortico-thalamo-striatal loops, propagating information mostly from a ventral to a dorsal direction (Yin and Knowlton, 2006). Despite the recent suggestion that the more dorsal mPFC handles more abstract and complex information than the more ventral mPFC (Denny et al., 2012; Suzuki et al., 2012), the specific roles of distinctive mPFC subregions and the exact nature of the interaction between them in the service of social behavior are currently unknown. In the next section, I will review the empirical and theoretical works on the functional properties of each subregion in more detail before proposing an integrative model of the mPFC function.

### Role of the Ventromedial Prefrontal Cortex (vmPFC) in Social Valuation

Many animal and human studies have described various aspects of the function of the vmPFC, including the regulation of emotions (Quirk and Beer, 2006; Milad et al., 2009; LaLumiere et al., 2010; Delgado et al., 2016), valuation for decision-making (Kable and Glimcher, 2007; Kim et al., 2007; Bartra et al., 2013), and goal-directed actions (O’Doherty, 2011). The vmPFC is also thought to be a key cortical component of the central autonomic network (Beissner et al., 2013). Consistent with this, there is additional evidence that the vmPFC may be involved in encoding internally driven valuation. For example, the vmPFC is the main target of the dopaminergic projection from the midbrain modulated by either food intake (de Araujo et al., 2012) or direct vagus nerve stimulation (Han et al., 2018). The vmPFC activity is also modulated by the experimentally-induced dopamine level (Jocham et al., 2011), visceral signals like hunger and satiety (Roy et al., 2012; Howard et al., 2015), and outcome devaluation (Valentin et al., 2007; de Wit et al., 2009). Also, vmPFC activity covaries with heart rate variability (Ziegler et al., 2009), and vmPFC lesions impair expression of normal physiological responses during decision-making (Bechara et al., 1996).

The vmPFC is known to have dense anatomical connections with both the nucleus accumbens (Haber et al., 2006) and the amygdala (Carmichael and Price, 1995; Ghashghaei et al., 2007). The former is mainly involved in learning reward-seeking behavior by reinforcing actions to obtain reward (Ikemoto and Panksepp, 1999; Demos et al., 2012; Lawrence et al., 2012), whereas the latter is primarily involved in learning defensive behaviors to avoid potentially dangerous or unpleasant stimuli (Schwartz et al., 2003; Mason et al., 2006; De Martino et al., 2010). These connections may allow the vmPFC to generate rapid avoidance or approach responses, making it an ideal system for a cost-benefit analysis to achieve homeostatic balance in a given situation (Schneirla, 1959; Kim et al., 2006; Basten et al., 2010).

In the field of social neuroscience, the vmPFC has been strongly implicated in processing “first-person” information (Kim et al., 2007; Denny et al., 2012; Bzdok et al., 2013; Lebreton et al., 2009; Levy et al., 2011), although several studies have also shown that the vmPFC activity can be commonly involved in decisions for both self and others (Nicolle et al., 2012; Janowski et al., 2013). Such an inconsistency can be reconciled by assuming the role of the vmPFC function in intuitive, internalized valuation for other-regarding decisions. Supporting this idea, the vmPFC was shown to be involved in decision-making for others, when people apply self-simulation to estimate a stranger’s preferences (Janowski et al., 2013; Kang et al., 2013) and when people are fully familiarized with others’ preferences through practice (Nicolle et al., 2012). These findings, therefore, indicate that the vmPFC could be involved in computing the value of choices for others, only when such valuation process is internally driven via familiarization of others’ preferences or through egocentric simulations.

Similarly, in the specific context of prosociality, the vmPFC seems to encode decision values for highly internalized forms of altruistic behaviors (i.e., internalized prosocial valuation) as in harm-aversion in social dilemma and moral emotions (Moll et al., 2006; Hare et al., 2010; Shenhav and Greene, 2010; Tricomi et al., 2010; Zaki and Mitchell, 2011; Buckholtz and Marois, 2012; Crockett, 2013; Sul et al., 2015). For example, a more recent study showed that selfish people used the vmPFC only when calculating the value of the choices for themselves but not those for strangers, unlike altruistic people who used the vmPFC for both self and other (Sul et al., 2015). Besides, more prosocial people showed higher vmPFC activity during prosocial choice, whether they are observed by others or not, and higher vmPFC activity was associated with faster response time for prosocial choices (Jung et al., 2018). Taken together, these findings suggest that prosocial valuation encoded by vmPFC may be intuitively engaged and immune to social context.

According to recent theories on morality and altruism (Haidt, 2007), the ultimate desire for survival and reproduction can be extended to creating an altruistic instrumental desire to sacrifice oneself for others. That is, people can learn the belief that the act of helping others is an effective way to draw a favorable impression from others, and such a belief can be internalized to create a new instrumental desire. Such an instrumental desire for altruism may be internalized in the vmPFC, which may then facilitate prosocial behavior automatically and intuitively, more or less independently of social context (Rand et al., 2012; Sul et al., 2015; Jung et al., 2018). This idea is also consistent with the findings that the vmPFC is associated with seeking social status (Milad et al., 2009; Hughes and Beer, 2012, 2013). For example, an altruistic decision may result from the motivation to avoid the possibility of losing reputation due to selfish behavior. In this sense, the vmPFC activity associated with prosocial behavior may indicate the degree to which one’s valuation for social reward is internalized, and, therefore, is resistant to contextual changes.

### Role of the Dorsomedial Prefrontal Cortex (dmPFC) in Social Valuation

The dorsomedial prefrontal cortex (dmPFC) has been implicated in numerous aspects of psychological functions (Ebitz and Hayden, 2016), such as detecting and resolving conflicts among competitive responses (Shenhav et al., 2016), searching for a new value beyond the current familiar state (Kolling et al., 2016), and computing decision values based on external sensory signals from the environment, unlike the vmPFC involved in internal valuation (Bouret and Richmond, 2010; Nakao et al., 2012; Howard et al., 2015). Consistent with the functional dissociation between dmPFC and vmPFC in humans, recent studies on rats have also shown a functionally competitive relationship between dorsal and ventral subregion of the mPFC (Coutureau and Killcross, 2003). For example, the prelimbic cortex, a more dorsal part of the mPFC in rats comparable to BA 32 in primates (Vogt et al., 2013), is responsible for voluntary and goal-directed initial responses, whereas the infralimbic cortex, a more ventral part of mPFC in rats comparable to BA 25 in primates, is responsible for developing habit-like behaviors, which are formed progressively through overtraining (Killcross and Coutureau, 2003). In addition, the BA 32 along with its neighboring cortical regions including the rmPFC or the dmPFC have heavy anatomical connections with the hippocampus and the related rhinal cortex (Barbas, 2015), and lesioning these cortices resulted in significant impairment in the mnemonic retrieval of context within which external sensory stimuli are experienced (Chapados and Petrides, 2015). Based on these findings, it can be speculated that, when two or more competing responses come into conflict, the dmPFC is engaged to search for a new and more appropriate response to resolve the conflict by directing attention to external sensory information from the environment or information available in memory (Cabeza et al., 2002; Horst and Laubach, 2009), which may have little to do with fulfilling the immediate internal needs of the body.

The dmPFC has been often shown to respond to negative outcomes such as pain (Rainville et al., 1997), monetary loss (Liu et al., 2011), as well as social rejection (Eisenberger et al., 2003). Some recent theoretical works also suggested a more general function of the dmPFC, that is, to integrate multiple sources of information from a wide range of brain network to guide our thoughts and actions (Shackman et al., 2011), or to maintain the representation of expected reward and to allocate available physiological resources to meet or exceed task demands (Touroutoglou et al., 2020). One can speculate that experiencing negative outcome may trigger neural processes of re-allocating attention to the environment in order to search for a new potentially better alternative, whereas experiencing positive outcome may elicit a simpler strategy of maintaining previously chosen behavior that have led to the successful consequence. Consistent with the evolutionarily advantageous decision heuristic of *win-stay lose-shift* (Nowak and Sigmund, 1993), this view suggests that positive and negative outcomes are naturally associated with internal and external valuation process, which are mainly subserved by the vmPFC and the dmPFC, respectively.

In social neuroscience, contrary to the role of the vmPFC in processing “first-person” information, the dmPFC appears to be more involved in processing “third-person” information, which includes mentalization or perspective-taking (Amodio and Frith, 2006; Frith and Frith, 2006; Mitchell et al., 2006; Hampton et al., 2008; Behrens et al., 2009; Kang et al., 2013), valuation of decisions for others (Suzuki et al., 2012; Jung et al., 2013; Hutcherson et al., 2015; Sul et al., 2015), evaluation of outcomes given to others (Chang et al., 2013; Apps and Ramnani, 2014; Lockwood et al., 2015), and prosocial behavior (Waytz et al., 2012). Despite these other-centered functions, the dmPFC activity is not always associated with prosocial behavior. For example, the dmPFC activity encoding value of decision for others was more prominent among selfish compared to prosocial people (Sul et al., 2015), and the value-related dmPFC activity was stronger for self-centered than other-oriented decisions under social observation (Jung et al., 2018). These inconsistencies about the role of the dmPFC in prosocial decisions should be examined more carefully by considering the differences among studies in the experimental context. Given that the dmPFC is also associated with strategic decisions that maximize profits (Rilling et al., 2004; Hampton et al., 2008; Behrens et al., 2009; Seo et al., 2014), it can be inferred that the dmPFC activity can lead to prosocial behavior only when such deliberate decisions regarding others are strategically beneficial to decision-makers.

Taken together, these findings suggest that the dmPFC activity may predict prosocial behavior only when the context intuitively triggers selfish behavior, however, prosocial behavior can be strategically more beneficial. Conversely, the same region may be engaged even when the context automatically triggers prosocial motivation, however, economic value maximization can be strategically more beneficial. Therefore, the dmPFC can be engaged whenever a conflict occurs among two or more responses, and consideration of additional (external) information is necessary for value-maximization, regardless of whether its activity leads to a prosocial outcome or not.

### Role of the Rostromedial Prefrontal Cortex (rmPFC) in Social Valuation

The rmPFC, which lies between the vmPFC and the dmPFC, has unique and privileged anatomical features because of its widespread anatomical connections with many cortical and subcortical structures including the brainstem, the insula, and most of the other mPFC subregions (Dixon et al., 2017). This region has been implicated in various functions such as default-mode processing (Uddin et al., 2009; Andrews-Hanna et al., 2010), far-sighted decisions, where one needs to choose between immediate smaller and delayed more substantial reward (Kable and Glimcher, 2007), and, most notably, cognitive branching, that is, pursuing a long-term mental plan by tracking the values of ongoing and alternative behavioral strategies and switching to the better option (Koechlin and Hyafil, 2007; Mansouri et al., 2017).

In social neuroscience, the rmPFC has been best known for its prioritized role in self-referential processing (Kelley et al., 2002; Moran et al., 2006; Northoff et al., 2006), although it also has been shown to encode decision values for both self and others (Hutcherson et al., 2015; Sul et al., 2015). For example, in a typical self-referential task where participants view a list of trait-related words and report whether they are self- or other-descriptive, increased activity is found in the rmPFC during conditions of self vs. other (Kelley et al., 2002). Different groups of researchers have interpreted such a self-referential activity in the rmPFC as perceived similarity (Mitchell et al., 2006), personal significance (Krienen et al., 2010; Kim and Johnson, 2015), and social valuation (D’Argembeau, 2013). An alternate, possibly more plausible, reason for the rmPFC activity during a self-referential task might be that it reflects heightened motivation for seeking self-enhancement, including both self-promotion (approach) and self-protection (avoidance), which is similar to its suggested role in reputation management (Amodio and Frith, 2006; Izuma, 2012). According to this account, the rmPFC activity increases during self- vs. other-referential task because one feels a greater need to engage motivation for self-enhancement. This alternative view can be supported by several recent findings listed below.

First, the rmPFC activity is often associated with different types of self-conscious emotions that can occur depending on whether one’s behavior is appropriate to social standards or not (Edelmann, 1987; Leary and Kowalski, 1990; Keltner and Buswell, 1997; Tracy and Robins, 2004; Tangney et al., 2007). For example, in many studies using emotion-evoking scenarios, rmPFC activity has been linked to subjective experience of various self-conscious emotions such as embarrassment (Takahashi et al., 2004; Burnett et al., 2009; Bas-Hoogendam et al., 2017), shame (Michl et al., 2012), guilt (Shin et al., 2000; Takahashi et al., 2004; Zahn et al., 2008; Burnett et al., 2009; Basile et al., 2011; Wagner et al., 2011; Fourie et al., 2014; Gilead et al., 2016), and pride (Zahn et al., 2008; Gilead et al., 2016).

Second, the structural and functional integrity of the rmPFC is linked to individual differences in motivation for self-enhancement. For example, people with high rejection sensitivity showed increased rmPFC activity when anticipating social evaluation (Powers et al., 2013), and those with high trait social anxiety are characterized with heightened rmPFC activity during social observation (Müller-Pinzler et al., 2015). Also, patients with lesions in areas including rmPFC failed to exhibit self-conscious emotion (Sturm et al., 2006, 2008; Krajbich et al., 2009; Moll et al., 2011) and expressed socially inappropriate self-disclosing behavior (Beer et al., 2006), and those with smaller volume of rmPFC showed reduced physiological and behavioral indices of self-conscious emotional responses when watching a video clip of themselves singing (Sturm et al., 2012). Recently, it was also demonstrated that the developmental maturity of the rmPFC may be critical for a more sophisticated and socially appropriate expression of self-protective motivation in response to negative evaluation from others (Yoon et al., 2018).

**FIGURE 2:**
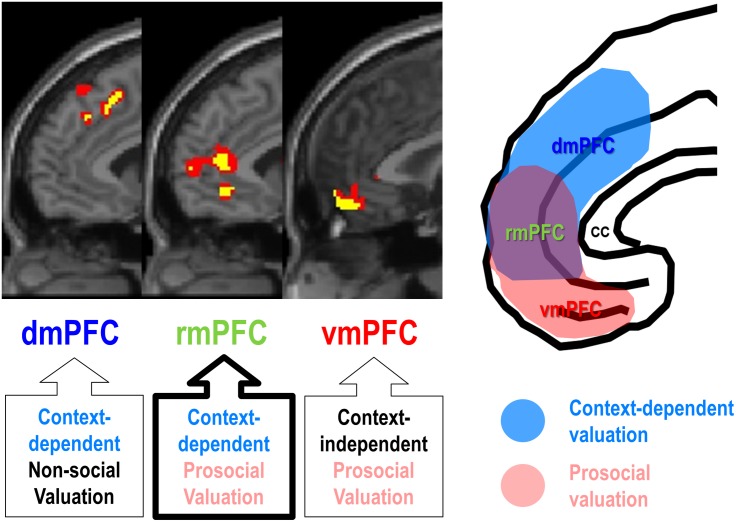
Functional segregation of mPFC function during ethical consumption under social observation. The rmPFC and the dmPFC encode subject-specific values of purchasing social products (prosocial decision) and non-social products (self-centered decision), respectively, under social observation (context-dependent), whereas the vmPFC encodes subject-specific values of purchasing social products regardless of social observation (context-independent) (Adapted from Jung et al., 2018).

Third, social observation, one of the most potent situational factors boosting self-enhancement motivation, can modulate the rmPFC activity, often leading to increase in a socially desirable behavior. For example, social observation increased rmPFC activity during judgment about self and social appropriateness (Izuma et al., 2010), public success or failure on a cognitive task (Müller-Pinzler et al., 2015), and an economic game (Van Hoorn et al., 2016), often being accompanied by subjectively experienced self-conscious emotion (Somerville et al., 2013). Consistent with the “costly signaling theory,” which views altruistic or prosocial behavior as a signal of willingness and ability to help others (Zahavi, 1975; Nowak and Sigmund, 1998; Fehr and Fischbacher, 2003; Hardy and Van Vugt, 2006), social observation by others or even subtle surveillance cues can be powerful enough to increase prosocial behavior (Soetevent, 2005; Bereczkei et al., 2010; Griskevicius et al., 2010; Izuma, 2012; Kimura et al., 2012). In line with the behavioral evidence of costly signaling theory, social observation also increased the rmPFC activity encoding the value of prosocial decisions, and such a context-dependent prosocial valuation of the rmPFC was clearly distinguishable from those of the vmPFC and the dmPFC (Figure 2; Jung et al., 2018). It should be noted, however, that context-dependent functionality of rmPFC for social valuation does not necessarily require social observation. For example, increased rmPFC activity was associated with strategic prosocial behavior when participants had been explicitly instructed to make a donation with money endowed by the experimenter, which may have posed a substantial social pressure, similar to a social observation (Tusche et al., 2016; Cutler and Campbell-Meiklejohn, 2019; Fukuda et al., 2019).

Lastly, the rmPFC integrates social information to update the subjective estimation of self-efficacy or social status under competitive or cooperative social contexts. For example, several recent studies have shown that the rmPFC activity tracks trial-by-trial fluctuation of expected social dominance during competitive perceptual decision tasks (Ligneul et al., 2016), dynamic updates of self-efficacy, estimated based on self and other’s performances in perceptual decision task (Wittmann et al., 2016), prediction error signals between expected and observed social feedback from others (Korn et al., 2012; Will et al., 2017), updating knowledge of one’s own social hierarchy (Kumaran et al., 2016), and value of self-protective behavior in response to negative social feedback (Yoon et al., 2018). According to a recent hypothesis, self-efficacy can be metacognitive beliefs about the brain’s capacity to successfully regulate bodily states and the rmPFC plays a crucial role in such “allostatic self-efficacy” (Wager et al., 2009; Stephan et al., 2016). Taken together, these studies indicate that at the core of self-enhancement motivation lies the allostatic regulatory function of the rmPFC, that is, shaping the internal drive for (bodily) homeostasis so that it better fits into the constraints of external (environmental) contextual variables (Smith et al., 2017).

In summary, under situations where one’s impression or reputation is at stake, the rmPFC may arbitrate between intuitive motivation for self-enhancement (i.e., internal valuation) and careful consideration of contextual information (i.e., external valuation). Such an rmPFC arbitration function may be critical for the neural mechanism of allostatic regulatory control that serves to meet internal bodily needs in a socially relevant manner.

In the next section, I will introduce the hierarchical allostatic regulation model of mPFC function and show how hierarchically organized subregions of mPFC can interact with each other in such a way that more dorsal regions utilize additional external information from environment to predict and prevent conflicts occurring in more ventral regions tuned to internal bodily signals. In the end, I will show how such a hierarchical allostatic regulatory mPFC function can effectively address the stability-plasticity dilemma in a constantly changing social environment.

## Hierarchical Allostatic Regulation Model of mPFC Function for Social Valuation

### mPFC Subregions Encoding Gradient of Internal-to-External Valuation

In the hierarchical model of social valuation, the mPFC comprises three functionally dissociable and hierarchically organized subregions: vmPFC, rmPFC, and dmPFC. These regions are differentially involved in computing values of decision along the ventral-to-dorsal spatial gradient of increasing external sensory inputs (e.g., via the temporal cortex and the parietal cortex) and decreasing internal inputs (e.g., via the brainstem, the hypothalamus, the amygdala, and the nucleus accumbens) (Dixon et al., 2017). Such an mPFC functional gradient is also consistent with the direction of evolutionary progress revealed by a recent analysis of the sulcal organization pattern across primate species (Amiez et al., 2019).

It should be noted, however, that the boundary between internal and external valuation is only a relative one. A level can be either internal or external depending on whether it is compared with its upper or lower level, respectively. For example, the vmPFC can be an internal valuation system when compared with the rmPFC but can also be an external valuation system when compared with the amygdala and the nucleus accumbens. In addition, the internal valuation of the vmPFC should be distinguished from instinctive or reflex-like responses that may be controlled by other structures, such as the amygdala, nucleus accumbens, and the spinal cord (located at a level further below the vmPFC). Therefore, the vmPFC can also be involved in valuation that requires abstract and sophisticated representations of a task structure if such representations became internalized through repetition (Hampton et al., 2008) as well as in goal-directed decisions when goals are primarily determined by internal signals carrying a homeostatic bodily state (Valentin et al., 2007).

**FIGURE 3:**
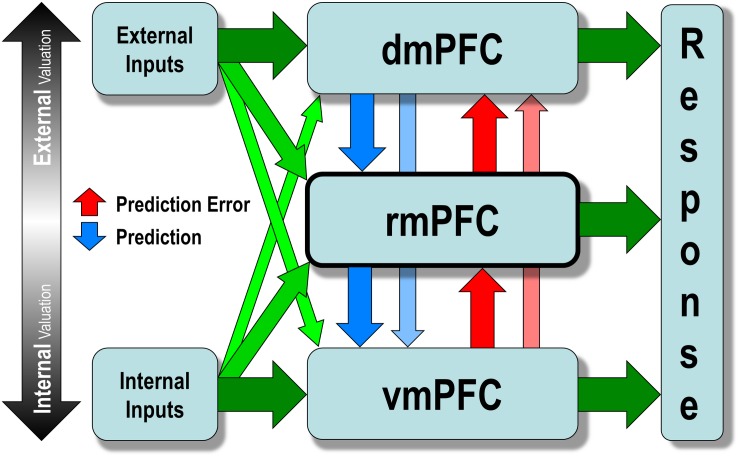
The hierarchical allostatic regulation model of mPFC function for social valuation. The mPFC comprises of three functionally dissociable hierarchically organized subregions: the vmPFC, rmPFC, and dmPFC, which are differentially involved in computing values of decision along the ventral-to-dorsal spatial gradient of increasing external sensory inputs (e.g., via the temporal cortex and the parietal cortex) and decreasing internal inputs (e.g., via the brainstem, the hypothalamus, the amygdala, and the nucleus accumbens). The vmPFC computes the *internal valuation* that generates interoceptive prediction signals and elicits a familiar intuitive response to prevent foreseen bodily imbalance. When two or more mutually incompatible values are simultaneously activated at the level of vmPFC, a conflict (prediction error) may occur triggering the upper levels (i.e., either rmPFC or dmPFC), which would then disengage internal valuation and increase the sensitivity to incoming sensory signals from the external environment to resolve the conflict. Such a process, called *external valuation*, sends prediction signals to update the preexisting value encoded at the lower level and continues until it finds a new value that resolves the conflict. The newly updated value will be strengthened and internalized through repetition so that it is activated more quickly and easily in similar situations later without causing a conflict.

Briefly speaking, the model detects prediction error in the internal valuation at the lower level and triggers the external valuation at the upper level to update the preexisting values at the lower level. Both external and internal input can trigger internalized values encoded by the vmPFC, which then activate interoceptive prediction signals that trigger a familiar, intuitive, and habit-like response to prevent anticipated bodily imbalance. Such a process is called *internal valuation*. However, when two or more mutually incompatible values are simultaneously activated at the level of the vmPFC, a conflict (i.e., prediction error) occurs, which then disengages the internal valuation and engages the upper levels (i.e., either rmPFC or dmPFC). The upper levels would then engage in resolving the conflict at the lower level by increasing sensitivity to incoming sensory signals from the external environment, taking over the decision control temporarily by searching for a new and more sophisticated stimulus-response mapping. Such a process, called *external valuation*, sends prediction signals to update the pre-existing mapping at the lower level, and continues until it finds a new mapping that resolves the conflict. The new mapping will be strengthened and internalized through repetition so that it is activated quicker and more comfortably in similar future situations without causing a conflict (Figure 3).

This model naturally predicts that easy decisions should activate the vmPFC due to a weak conflict between options, whereas difficult decisions should elicit the dmPFC due to a strong conflict between options with similar value. Supporting this prediction, larger and smaller differences in value between two alternative options in a choice task were associated with greater activity in the vmPFC and the dmPFC, respectively (Hackel et al., 2017; Piva et al., 2019). According to the model, in the case of larger value difference, the vmPFC alone can handle the value computation for decision, whereas, in the case of smaller value difference, the dmPFC needs to be engaged to integrate additional information from the environment to resolve the conflicts in the vmPFC.

To illustrate better how the model works, especially in the social domain, prosocial behavior can be a good example. If the value for a prosocial decision does not conflict with the value for a self-interested decision, then the prosocial behavior can be triggered rapidly via the intuitive internal valuation by the vmPFC. However, in cases where the two values conflict with each other in the vmPFC, additional information must be considered to resolve the conflict and to choose a more appropriate value in each context or to create a new value as a more appropriate alternative. By incorporating increasingly complex external information, the process of creating more efficient and sophisticated behavioral rules can produce various abstract social values, which can later be internalized through repetition. For example, an infant may learn to attract the attention of his/her caregiver to keep the caregiver closer. Such behaviors are often reinforced by the successful avoidance of hunger and insecurity. These primary forms of social reward can later serve as powerful internalized motivation for making friends and pursuing social status as a child grows up. Possibly, various secondary reinforcers, such as money and social reward, are learned because they serve the common goal of preventing anticipated homeostatic imbalance (e.g., hunger or pain), and these newly acquired values can be internalized in the vmPFC to enable rapid comparisons between various types of reward, serving as the common neural currency (Izuma et al., 2008; Chib et al., 2009; Kim et al., 2011; Levy and Glimcher, 2012; Lin et al., 2012).

### Role of the Thalamic Reticular Nucleus in Switching Between Thalamo-Cortical Loops

As mentioned above, a successful adaptation in a continually changing environment would require a careful assessment of the efficiency of the currently engaged decision system as well as a flexible transition between intuitive (internal) and deliberative (external) decision systems. What makes our brain achieve such an elegantly complex adaptive function? One potential neural candidate that is crucial for such functions may be the thalamic reticular nucleus (TRN). The TRN consists of a layer of inhibitory neurons surrounding the thalamic nuclei, and can be divided into several sectors connected to different thalamic nuclei and their associated cortical regions, and is believed to serve as a nexus that moderates the interaction between separate sectors of thalamo-cortical loops and controls the transition between distinctive attentional modes (Crick, 1984; Guillery et al., 1998; Pinault, 2004). The TRN neurons exert an inhibitory control not only on the thalamo-cortical neurons but also on local inhibitory interneurons, which may lead to the disinhibition of the thalamo-cortical projection neurons (Steriade et al., 1985). The connection between the TRN neurons and the local inhibitory cells in the thalamus is believed to be subservient to the processes for focusing attention to relevant signals by suppressing other competing sub-networks processing non-relevant signals (Steriade, 1999), making the TRN neurons capable of detecting changes in the environment and modulating excitability of their target thalamic relay neurons (Yu et al., 2009).

The TRN neurons can be segregated into at least two functionally and anatomically distinctive groups: the anterior (or limbic) TRN sector and the posterior (or sensory) TRN sector, which have distinct connectivity patterns (Zikopoulos and Barbas, 2012) and are shown to control different behavioral states (Halassa et al., 2014). The former has dense connections with the limbic regions, including the vmPFC and the amygdala, whereas the latter is connected more strongly with the sensory cortices and corresponding thalamic nuclei (Zikopoulos and Barbas, 2006, 2007, 2012), possibly being involved in focusing attention to internal signals and external stimuli, respectively (Zikopoulos and Barbas, 2012; Halassa et al., 2014).

How could the TRN contribute to the transitions between internal and external processing? First, the TRN may be capable of hijacking on-going behaviors and mediates a rapid transition from the external (dorsal) to the internal (ventral) thalamocortical sector. More specifically, the internal sector can be engaged by default to address internal signals carrying information on anticipated homeostatic imbalance, and any sudden transition from an external sector to an internal sector appears to be driven by inputs from other internal sectors that are located even closer to the root of the hierarchical structure. Supporting this idea, the anterior sector of the TRN can be quickly engaged by the inputs from the amygdala and the vmPFC (Zikopoulos and Barbas, 2012). Such input signals can allow the TRN to interrupt and control on-going exploratory behaviors or external sensory processing and to initiate a rapid switch to the internal thalamocortical sector, which can then trigger stereotypical instinctive or reflexive behavioral responses that had been engaged repeatedly to deal with previous similar situations.

**FIGURE 4:**
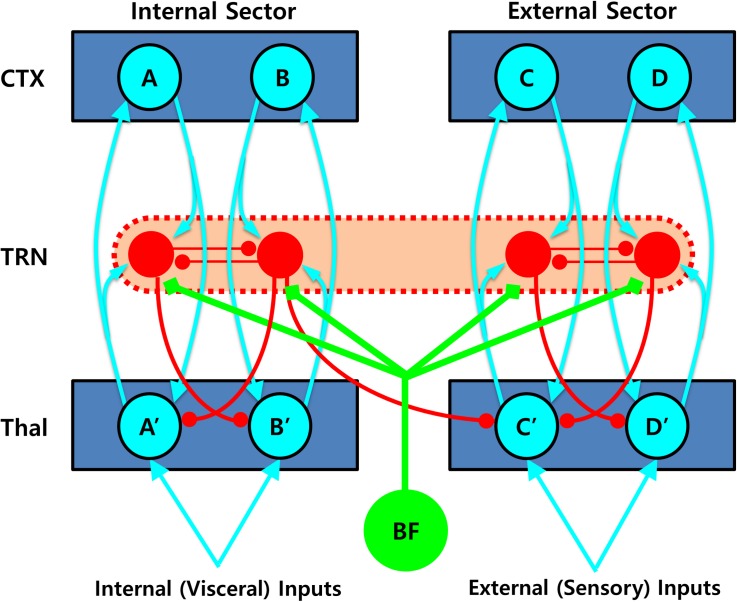
Role of the thalamic reticular nucleus (TRN) in shifting between the thalamo-cortical loops. Mutually inhibiting interneurons densely distributed in the TRN are perfectly suited for detecting conflicts between non-compatible units being engaged simultaneously, which can lead to disinhibition of other non-occupied units in the external sector, resulting in an attentional shift between different thalamo-cortical loops. In this diagram, simultaneous activation of two mutually competitive units (i.e., A and B) in the internal sector would lead to a sector-wide disinhibition of the thalamic projection neurons (i.e., C′ and D′) in the external sector, which would then gate the external sector, initiating more elaborated processing of additional external sensory information. Such inhibitory connections are likely to be asymmetrical, that is, favoring the direction from internal to external sectors and therefore prioritizing internal over external valuation. Note that not all the necessary connections are shown for visualization purpose. CTX, cortex; TRN, thalamic reticular nucleus; Thal: thalamus; BF: basal forebrain.

Second, the TRN is also capable of switching between thalamocortical sectors in the opposite direction, that is, from internal to external sectors. Such shifts can be achieved by the densely distributed inhibitory neurons in the TRN, which are responsible for lateral inhibition between different thalamocortical loops via direct mutual inhibition or inhibitory projection to the thalamic projection cells (John et al., 2016; Crabtree, 2018). Therefore, these neurons are ideally suited for detecting conflicts between simultaneously activated competing units in the internal sector of the TRN, which can lead to disinhibition of other non-occupied units in the external sector of the TRN network. To better illustrate the complex dynamics of the TRN network, a simplified diagram of the TRN networks is shown in Figure 4.

For example, simultaneous activation of two mutually competitive units (i.e., A and B) in the internal sector could lead to a sector-wide disinhibition of the thalamic projection neurons (i.e., C′ and D′) in the external sector. Such a disinhibition would then *gate* the external sector, initiating more elaborated processing of additional external sensory information. Considering that intra-TRN connections are mainly formed by gap junctions (Hou et al., 2016), the inter-sector inhibition could be best achieved by multiple divergent inhibitory projections from the TRN cells to the thalamic projection cells (Crabtree, 2018). Supporting this idea, such an “open-loop” TRN network, wherein a thalamic projection neuron is inhibited by the TRN neuron excited not by itself but by another thalamic projection neuron, has been identified in anatomical studies (Pinault and Deschenes, 1998; Kimura et al., 2007), and demonstrated to play a major role in signal propagation across distinct thalamocortical loops in a recent computation modeling study (Brown et al., 2020). It is noteworthy that such inter-sector inhibitory connections are assumed to be largely asymmetrical, that is, favoring the direction from internal to external sectors and therefore prioritizing internal over external valuation, consistent with the proposal that information propagates preferentially from a ventromedial to a dorsolateral direction across the thalamo-cortico-striatal loops (Yin and Knowlton, 2006). Although this is a speculative hypothesis that needs more concrete additional evidence, such an anatomical structure of the TRN network may be suitable for the mechanism of switching from the ventral (or internal) to the dorsal (external) thalamocortical sector that is functionally equivalent to the transition from an intuitive to a deliberative decision mode.

### Factors Affecting the Optimal Balance Between Stability and Plasticity

In the model mentioned above, the external valuation process in the upper level continues until the conflict is resolved at the lower level. Thus, it is reasonable to question how much conflict in the lower level is large enough to trigger the external valuation process, and to what degree the conflict needs to be resolved before disengaging the external valuation process. Answers to these questions are essential to understand how our brains deal with the challenging problem of the stability-plasticity dilemma. That is, any adaptive agent is expected to effectively address the stability-plasticity dilemma (Grossberg, 2013) to avoid either catastrophic forgetting (i.e., extreme plasticity) (Ratcliff, 1990) or the entrenchment effect (i.e., extreme stability) (Zevin and Seidenberg, 2004). For the optimal balance between stability and plasticity, therefore, an agent needs to carefully determine the optimal level of tolerance for mismatches between incoming sensory inputs and previously learned categorical representation. However, an agent often fails to maintain the balance due to the lack of information about the ultimate goal of an organism, which has to do with how precisely a prediction or model generated at the moment matches forthcoming internal and external states (Friston, 2010).

Pursuing the optimal balance between stability and plasticity can be related to the concept of *allostasis*, which allows an organism to produce system-wide behavioral and physiological adjustments to environmental challenges through prediction in advance of a need (Sterling and Eyer, 1988; McEwen and Stellar, 1993; Schulkin, 2003). Several recent theories suggest that the mPFC along with the insula form a neurocircuitry thought to convey allostatic predictions that modulate the set points of homeostatic reflexes (Critchley and Harrison, 2013; Sterling, 2014; Barrett and Simmons, 2015; Stephan et al., 2016; Kleckner et al., 2017), possibly via rapid direct or indirect communications with the brainstem (Allman et al., 2010; Fischer et al., 2016). Perhaps, the role of the mPFC in allostasis can be best understood by examining this region as one of the key cortical substrates for heart-rate variability (HRV) (Thayer et al., 2012). HRV is known to reflect the heart’s ability to detect and respond adaptively to unpredictable environmental changes and is regarded as a possible indicator of allostatic capacity to integrate behavioral strategies and energy stores in response to environmental demands (Grossman and Taylor, 2007).

Consistent with the hierarchical allostatic regulation model of mPFC function proposed in the present study, different mPFC subregions may be involved in distinctive aspects of HRV-related functions. For example, the vmPFC activity is known to covary with HRV, and the rmPFC is suggested to play a regulatory role over the autonomic response initiated by the vmPFC (Ziegler et al., 2009). This idea is further supported by a recent meta-analysis showing that the vmPFC and the rmPFC are involved in sympathetic and parasympathetic processes, respectively (Beissner et al., 2013). In line with this, a recent finding showed that pharmacological inactivation of the vmPFC and the rmPFC led to decreased and increased autonomic and behavioral responses, respectively, to negative emotional stimuli in non-human primates (Wallis et al., 2017). These findings suggest that, while the vmPFC quickly elicits familiar, internalized responses to cope with anticipated physical consequences of external stimuli, the rmPFC appears to seek a more holistic solution for harmonization between the fast-autonomic response and the constraints of the external environment.

Perhaps, another key contributor to the optimal balance between stability and plasticity may be the neuromodulatory afferent signals targeting the allostatic neurocircuitry mentioned earlier as well as the TRN network. For example, there is evidence that the insula, the mPFC, and the TRN are the major targets of the cholinergic signals from the basal forebrain (Haber and Calzavara, 2009). In general, these neuromodulatory signals can potentiate presynaptic glutamatergic and GABAergic neurotransmission (Freund et al., 1988; Alkondon et al., 1997; Radcliffe et al., 1999). These signals can facilitate the competition between simultaneously activated mutually incompatible units, leading to an enhanced signal-to-noise ratio in the target area (Everitt and Robbins, 1997; Sarter and Bruno, 1997). Therefore, such cholinergic neuromodulatory inputs to the TRN inhibitory network could increase the sensitivity to the conflict in the internal sector as well as the likelihood of disinhibiting otherwise suppressed units in the external sector, which could then lead to enhanced precision in the environmental sensory information (Feldman and Friston, 2010). Through this process, cholinergic signals can lower the degree of tolerance for a mismatch between actual and predicted bodily states, which can then lead to frequent and prolonged engagement of the external valuation process that will continue to search for a new categorical representation to resolve the mismatch. In this sense, the role of cholinergic signals in modulating competition in the TRN is analogous to the concept of *vigilance parameter* that determines the allowable degree of mismatch between any input pattern and any stored patterns, resulting in either crude (i.e., low vigilance) or fine (i.e., high vigilance) categorization of incoming stimuli (Grossberg, 2013).

In addition to cholinergic signals, some other neuromodulatory signals, such as dopamine and noradrenaline, have been shown to serve similar functions; that is, signaling the degree of sensitivity to the discrepancy between prediction (or belief) and actual sensory information (Friston et al., 2014). For example, the dopamine neurons in the midbrain signal the discrepancy between predicted and actual reward (Schultz, 1998), and the noradrenergic neurons in the locus coeruleus can interrupt the activity of on-going functional networks and facilitate their reorganization to promote rapid behavioral adaptation (Bouret and Sara, 2005). These different neuromodulatory signals may serve a common goal of adjusting the balance between stability and plasticity, by reporting an integrative sum of internal milieu or interoceptive prediction errors to the brain (Fadel and Burk, 2010). To this end, a higher sensitivity to interoceptive prediction errors can lead to a higher vigilance, resulting in finer mappings between internal needs and external environment, which may indicate a more adaptive capacity for allostatic regulation. As an example in the domain of social neuroscience, people with a polymorphism in the dopamine D4 receptor gene (*DRD4*), which is associated with a higher sensitivity to environmental reward, are more likely to display behaviors that are more culturally dominant and socially desirable, compared to those with other types of *DRD4* polymorphisms (Kitayama et al., 2014). In summary, different types of neuromodulatory signals may share a common goal of allowing the brain to continuously check and modulate the precision of interoceptive prediction and to build an accurate and sophisticated internal model of visceral states (Friston et al., 2014), which can be advantageous for pursing adaptive behavior in a constantly changing social environment.

### Self-Control, Self-Efficacy, and Metacognition as Types of Allostatic Regulation

Recent theories suggest that interoceptive prediction errors reporting homeostatic or allostatic imbalance are essential for valuation of decisions (Gu and FitzGerald, 2014) as well as cognitive and goal-directed control over habitual actions (Pezzulo et al., 2015). These theories can be further refined by considering the allostatic function of the rmPFC in solving the stability-plasticity dilemma. For example, people with high baseline HRV showed greater self-control during a food choice task and higher rmPFC activity when the participants had to overcome their taste preferences to choose the healthier option (Maier and Hare, 2017). Similarly, the rmPFC has also been shown to be involved in *meta-decision*, that is, choosing between distinctive decision systems. For example, the rmPFC is known to play a vital role in arbitrating the transition from the intuitive (model-free) to the analytical (model-based) decision systems, by tracking prediction error signals arising from the performance of the model-free system (Lee et al., 2014). Based on the model introduced above, such arbitration can be best explained by the function of the rmPFC in detecting and resolving conflict in internal valuation by referring to additional external information. When engaged, the rmPFC may quickly resolve the conflict in the vmPFC, or it may trigger an even higher level of external valuation computed by the dmPFC. The rmPFC may be particularly suitable for such a function of arbitration between internal and external valuation, because of its privileged anatomical feature of integrating balanced inputs from both internal and external sources of incoming information.

The rmPFC has also been implicated in metacognition. For example, the rmPFC has been shown to track changes in the subjective sense of decision confidence (Bang and Fleming, 2018), and lesions to the anterior sectors of the prefrontal cortex, including rmPFC, led to an impairment of perceptual metacognitive accuracy (Fleming et al., 2014). Similarly, the rmPFC function in context-sensitive reputation management (Jung et al., 2018) may reflect metacognitive monitoring of the appropriateness of intuitive and internalized valuation for self-enhancement under the constraints of social contexts, seeking the optimal decision via referring to interoceptive prediction error signals and engaging external valuation whenever necessary. From this point of view, the role of the rmPFC in seeking the balance between internal and external valuation, which may be critical for successful allostatic regulation, may also be the core neural foundation shared among various forms of adaptive functions such as self-efficacy, self-regulation, and metacognition (Zimmerman and Moylan, 2009).

## Conclusion

The present study proposes that the mPFC subregions are hierarchically organized and differentially involved in computing values of decision, forming the ventral-to-dorsal spatial gradient of increasing external sensory inputs and decreasing internal inputs and exerting the hierarchical allostatic regulatory control over homeostatic reflexes. This hierarchical allostatic regulation model of mPFC function also emphasizes the role of the TRN in arbitrating the transitions between functionally dissociable thalamo-cortical loops. Because of its unique anatomical architecture with a robust inhibitory network, the TRN is capable of rapid and powerful orchestration over multiple thalamo-cortical loops and is critical for detecting and resolving conflicts between available options for decision-making and in shifting flexibly between decision modes. Importantly, neuromodulatory afferents to the TRN, signaling overall misfit of the interoceptive prediction model, can modulate the degree of sensitivity to the mismatch between predicted and observed bodily states. Such modulatory signals are critical for the mPFC function in allostatic regulation, which seeks an optimal balance between stability and plasticity, which can serve to maximize the probability of survival. In conclusion, the present model of the mPFC function can provide a useful theoretical framework, whereby previous findings once scattered around the mPFC area can be incorporated to generate and test novel hypotheses. Furthermore, future studies focusing on the hierarchical nature of the mPFC function can further expand our knowledge in various clinical symptoms, such as addiction, anxiety, and depression, which may be caused by a failure of the key neural mechanism for dealing with the stability-plasticity dilemma.

## Author Contributions

The author confirms being the sole contributor of this work and has approved it for publication.

## Conflict of Interest

The authors declare that the research was conducted in the absence of any commercial or financial relationships that could be construed as a potential conflict of interest.

## Abbreviations

dmPFC: dorsomedial prefrontal cortex
HRV: heart-rate variability
mPFC: medial prefrontal cortex
rmPFC: rostromedial prefrontal cortex
TRN: thalamic reticular nucleus
vmPFC: ventromedial prefrontal cortex.
